# The Role of Background Activity Monitoring by Amplitude-Integrated EEG to Predict Short-Term Neurological Outcome in Neonates with Congenital Heart Disease: Insights from a Real-Life Retrospective Cohort

**DOI:** 10.3390/neurosci7020048

**Published:** 2026-04-20

**Authors:** Massimo Mastrangelo, Salvatore Mazzeo, Eleonora Ferrante, Giulia Bruschi, Gianni Cutillo, Elisa Bortolin, Alessandro Bombaci, Irene Borzillo, Giuseppe Isgrò, Massimo Chessa, Alessandro Giamberti, Marco Ranucci, Massimo Filippi, Maria Salsone

**Affiliations:** 1 Unit of Cardiovascular Anesthesia and Intensive Care, IRCCS Policlinico San Donato, 20097 San Donato Milanese, Italy; 2 Faculty of Medicine and Surgery, Vita-Salute San Raffaele University, 20132 Milan, Italy; 3 Neurology Unit, IRCCS Policlinico San Donato, 20097 San Donato Milanese, Italy; 4 Department of Psychology, Vita-Salute San Raffaele University, 20132 Milan, Italy; 5 Pediatric and Adult Congenital Disease Heart Centre, IRCCS Policlinico San Donato, 20097 San Donato Milanese, Italy; 6 Unit of Congenital Cardiac Surgery, IRCCS Policlinico San Donato, 20097 San Donato Milanese, Italy; 7 Neurology Unit, Neurorehabilitation Unit, and Neurophysiology Service, IRCCS Ospedale San Raffaele, 20132 Milan, Italy

**Keywords:** congenital heart disease (CHD), neonates, neuromonitoring, electroencephalography (EEG), amplitude-integrated EEG, background activity

## Abstract

Neonates undergoing surgery for congenital heart disease (CHD) are at high risk for brain function impairment. Reliable early predictors of postoperative neurological complications are lacking. We examined a retrospective cohort of 55 surgically treated CHD neonates systematically monitored by concomitant conventional electroencephalography (cEEG) and amplitude-integrated EEG (aEEG). Neonates underwent cEEG/aEEG at three time points: T0 (preoperative, duration: 90–120 min); T1 (24–48 h after cardiac surgery, duration: ≥11 h); and T2 (7–10 days post-surgery, duration: 90–120 min). For each patient, aEEG background activity was evaluated and scored, and clinical and surgical data were retrieved to establish short-term post-surgical outcomes. Patients with normal T0 monitoring had significantly higher aEEG bandwidths in T1. A lower Aristotle basic score was associated with an improvement in aEEG at T1. Inversely, a narrower aEEG bandwidth in T1 was associated with post-surgical neurological deterioration. The aEEG bandwidth accurately predicted short-term neurological outcome; in particular, a minimal aEEG amplitude above 17.5 µV excluded poor neurological outcome with a negative predictive value of 81.48%. Our results demonstrated that aEEG bandwidth and trend dynamics may be associated with surgical complexity and neurological outcomes. aEEG background trend monitoring may provide relevant prognostic information on neurological outcomes in surgically treated CHD neonates.

## 1. Introduction

There is robust evidence that neonates with surgically treated congenital heart disease (CHD) are at high risk for neurodevelopmental impairment [1]. Abnormal prenatal brain maturation, combined with perioperative and postoperative risks, can lead to functional deficits across cognitive, motor, and psychosocial domains, ultimately impacting quality of life [2]. Neurodevelopmental alterations in this population may often originate in utero. Impaired placental oxygen exchange leads to reduced fetal oxygenation, which contributes to delayed somatic and cerebral growth [3]. Additional prenatal factors (such as placental pathology, genetic and epigenetic variations, and pre- or perinatal fetal stress) further exacerbate cerebral vulnerability [4,5,6]. After birth, up to half of affected neonates exhibit preoperative brain injury on neuroimaging [1,7], and nearly one-third develop new or worsened lesions postoperatively [1,8]. Approximately 25–50% of neonates with CHD require cardiac intervention, with postoperative injuries most commonly manifesting as stroke or white matter injury [8]. These processes significantly increase the risk of epileptic seizures, which are among the earliest and most frequent clinical signs of neurological compromise and a recognized risk factor for neurodevelopmental deficits. Seizure incidence in neonates undergoing cardiac surgery ranges from 4 to 35% [2]. Given this vulnerability, accurate early neurophysiological assessment of brain activity is of critical diagnostic and prognostic importance. Conventional EEG (cEEG) remains the standard method for real-time evaluation of neonatal brain function. However, resource and time constraints restrict its availability, which has driven widespread adoption of amplitude-integrated EEG (aEEG) as a simplified bedside technique capable of monitoring background activity, sleep–wake cycles, and seizure detection, especially when combined with cEEG [9,10,11]. More importantly, recent consensus statements and studies indicate that aEEG features may correlate with neurodevelopmental outcomes [1,2,12]. Consequently, recent studies highlight an urgent need for a standardized postoperative EEG monitoring framework, supporting the integration of a multimodal neuromonitoring approach that could offer a more comprehensive physiological understanding [2,7], in line with real-world needs and availability. However, neurocritical care practices in neonates with CHD remain heterogeneous, and the value of different neuromonitoring approaches in predicting clinical and neurodevelopmental outcomes is still uncertain [1]. In the present study, we aimed to investigate whether early postoperative aEEG background trends are associated with short-term neurological outcome. Secondary objectives included exploring the relationship between postoperative aEEG trends and surgical complexity, as measured by the Aristotle basic score, and assessing whether baseline aEEG characteristics influence postoperative neurophysiological evolution. We hypothesized that early postoperative alterations in aEEG background activity may reflect perioperative cerebral vulnerability and, therefore, be associated with short-term neurological outcomes in this population.

## 2. Materials and Methods

### 2.1. Study Design

In the present study, we evaluated the clinical utility of aEEG background trend analysis obtained through combined cEEG–aEEG monitoring in neonates undergoing surgery for CHD. This study was designed as a retrospective observational cohort study conducted at the Neonatal and Pediatric Intensive Care Unit of IRCCS Policlinico San Donato (San Donato Milanese, Milan, Italy). Clinical, demographic, and neurophysiological data were retrospectively retrieved from institutional electronic medical records and the neurophysiology database. All EEG recordings were performed as part of routine clinical care within the neuromonitoring protocol adopted in our center.

### 2.2. Study Population

We included a consecutive series of neonates, defined as term infants from birth to 28 days of age or preterms up to 44 weeks post-conception, admitted to our center between December 2023 and September 2024 for preoperative care in anticipation of corrective cardiac surgery. All included neonates underwent combined cEEG/aEEG preoperatively, 24–48 h after cardiac surgery, and 7–10 days postoperatively. Demographic and clinical data collected included sex, gestational age, delivery mode, birth weight classified as appropriate for gestational age (AGA) or small for gestational age (SGA), APGAR scores, and presence of any associated syndromic conditions. Cardiac pathology, Aristotle score (which defines surgical complexity, ranging from 1.5 to 15) [13], need for extracorporeal membrane oxygenation (ECMO), occurrence of seizures and/or status epilepticus, sedative therapy, and neurological outcomes at discharge from intensive care or at the end of the neonatal period were also recorded.

### 2.3. Electroencephalographic Recording

Combined cEEG/aEEG and/or video EEG recordings were performed using portable Micromed^®^ Brain Quick systems, which allow simultaneous display of cEEG trace and two aEEG channels. cEEG tracings were acquired with standardized parameters, i.e., high-pass filter: 0.15 Hz; low-pass filter (antialiasing): 69.1 Hz; sampling rate: 256 Hz. Two aEEG tracings were derived from the cEEG recorded, specifically from the T4-C4 and T3-C3 channels, to ensure bilateral hemispheric coverage. Electrode placement followed the adapted International 10–20 System according to ACNS guidelines in neonates [14]. In addition to ECG, when available, polygraphic channels (electro-oculogram, mylohyoid polygraphy, pneumogram) were included to monitor background activity, sleep–wake cycles, and seizure occurrence. Artifacts were identified through simultaneous visualization in the monitoring screen of a combined cEEG/aEEG recording with the aid of polygraphic channels, which allowed improved recognition of artifacts that may affect the interpretation of aEEG traces. Segments with major artifacts were excluded from analysis. Moreover, ICU staff were trained in recognizing and, if feasible, correcting a range of common artifacts (e.g., problems with electrode placement, respirator disturbances, paroxysmal movements related to non-epileptic events). A complete description of the material used, channels, and their recording parameters is provided in the Supplementary Material.

Neonates were monitored at three defined timepoints. The first recording (T0) was performed at baseline, preoperatively, and lasted at least 90 min. The second (T1) was conducted within 24 hours and continued for not over 48 h after cardiac surgery, with a minimum duration of 11 h (range: 11–30 h). The third recording (T2) took place 7–10 days postoperatively and lasted at least 90 min. These time windows were chosen to represent clinically meaningful phases of perioperative management, namely the preoperative baseline (T0), the early postoperative phase characterized by hemodynamic instability and sedation (T1), and the early recovery phase approximately one week after surgery (T2). Additional recordings were performed as needed for abnormal EEG patterns, seizures, ECMO, low cardiac output, brain injury, or sedation at T2. For this study, the analysis focused on T1 recordings. Synchronized video, applied during the T0 and T2 sessions, allowed correlation of cerebral activity with behavior, artifact identification, and characterization of critical events. EEG recordings were performed without video in T1 due to post-surgical sedation of the patient.

### 2.4. Amplitude-Integrated EEG Classification

For the classification of the aEEG recordings at T0, T1, and T2, we applied an in-house developed classification system based on the previous literature [15,16,17,18,19,20] and summarized in Supplementary Table S1. In summary, key criteria for classification included the upper and lower margin amplitudes, overall bandwidth, lower margin variability, cyclicity, symmetry between hemispheres, and the occurrence of seizures/status epilepticus (Supplementary Table S1). aEEG was thus defined as: normal, moderately abnormal (further subdivided as moderately abnormal + or −), and severely abnormal.

Normal traces were characterized by an upper margin greater than 10 μV, a lower margin greater than 5 μV, a bandwidth below 15 μV during wakefulness or active sleep (with a lower margin > 5 μV) and below ≤50 μV during quiet sleep (with a lower margin > 5 μV), present and consistent cyclicity, present lower margin variability, absence of asymmetry, and absence of seizures. Traces classified as “Normal” also included those with minor anomalies insufficient to justify a lower classification, such as an isolated lower margin drop below 5 μV or a span that may occasionally exceed defined limits by less than 50%.

Traces classified as moderately abnormal plus (+) had an upper margin of at least 10 μV, and a lower margin of 5 μV or less. Since bandwidth span is not univocally defined in the literature, we considered reduced lower margin variability exceeding 5 μV, inconsistent or initial cyclicity, asymmetry that may be present or absent, and seizures that may be present but not consistent with status epilepticus diagnosis. Conversely, we classified traces as moderately abnormal minus (–) if they presented an upper margin above 10 μV and a lower margin of 5 μV or less, lower margin variability was absent or limited within the 0–5 μV range, absent cyclicity, asymmetry that may be present or absent (below 50% between the two hemispheres), and seizures that may be present but not consistent with status epilepticus diagnosis.

Severely abnormal traces exhibited an upper margin below 10 μV, a lower margin below 5 μV, a bandwidth span < 15 μV with a lower margin < 5 μV, or 20 μV with a lower margin < 5 μV, absent cyclicity, absent lower margin variability, asymmetry more than 50%, and seizures may be present and/or consistent with status epilepticus diagnosis. Severely abnormal traces included patterns such as burst suppression, characterized by an upper margin of >25 μV and a lower margin between 0 and 1 μV; continuous low-voltage tracing, where the lower margin remains consistently ≤5 μV and the upper margin is <10 μV; and isoelectric or flat tracing, where the band remains consistently below 5 μV.

**Figure 1:**
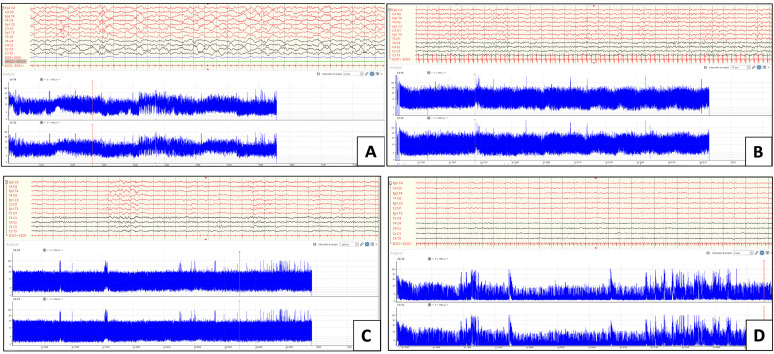
cEEG/aEEG epochs illustrating examples of the background across the proposed classification. All tracings were displayed with the following parameters: high-pass frequency filter = 1.6 Hz; low-pass frequency filter = 30 Hz; notch = active. Panel (**A**)—normal recording with variability and cyclicity of a newborn (38 weeks of corrected age [CE]). Panel (**B**)—moderately abnormal plus tracing of a 39-week-old CE newborn displaying initial lower margin variability, especially in the second half of the recording. Panel (**C**)—moderately abnormal minus tracing of a 39-week-old CE newborn displaying a single pattern with a lower margin below 5 µV and upper margin around 25 µV, without variability or cyclicity. Panel (**D**)—severely abnormal tracing of a 44-week-old CE newborn showing a persistently low voltage recording with a lower margin markedly below 5 µV and an upper margin below 10 µV. All tracings are displayed at a gain = 100 µV except for Panel D, which is displayed at 50 µV.

Examples of traces across the proposed classification are shown in Figure 1.

Then, for the purpose of the analysis, scorings were dichotomized as normal and abnormal (including moderately and severely abnormal). The monitoring trend was assessed by dividing the entire T1 recording (minimum period of 11 h) into three equal segments obtained by dividing the total recording time into thirds: initial (referred to as A), central (B), and final (C). Each segment was classified according to the above-mentioned criteria. Upper margin, lower margin, and bandwidth were recorded for each segment. Changes in scoring across the segments were then assessed, and aEEG monitoring trends were defined as improved if the classification changed from worse to better classification and not improved if the classification along monitoring remained the same or showed a worsening. cEEG/aEEG traces were visually reviewed and manually classified by a neurologist trained in pediatric and neonatal EEG (M.M.) and independently reviewed by an EEG technologist (E.F.) and a second neurologist (G.C.). Amplitude parameters were collected in mid-recording for each segment when the observed patterns are stable and artifact-free. Subsequently, all tracings were unblindly reviewed together, and a consensus was reached by discussion concerning lower and upper margins and the presence/absence of variability and asymmetry.

### 2.5. Neurological Outcome Classification

Neurological state at discharge from the ICU was classified by a neurologist trained in pediatric neurology (M.M.) as follows: (1) normal—clearly differentiated wake-sleep states, neutral posture in semi-flexion of limbs and/or spontaneous alternation of different postures, and well-differentiated motor activity; (2) moderately abnormal—reduced consciousness, limited postural variability, and reduced motor activity; and (3) severely abnormal—reduced consciousness, fixed-extended limb posture, and absent motor activity, coma or death during the observation period [21,22]. Neurological outcomes were then dichotomized as normal and abnormal (moderately and severely abnormal).

### 2.6. Statistical Analysis

Statistical analyses were performed using IBM SPSS Statistics Version 25 (SPSS Inc., Chicago, IL, USA) and the computing environment R ver. 4.5.2 (R Foundation for Statistical Computing, Vienna, 2013). Variable distributions were assessed using the Shapiro–Wilk test. Patient groups were described using medians and interquartile ranges (IQR) for continuous variables with non-normal distributions and using frequencies or percentages for categorical variables. The non-parametric Mann–Whitney U test was used for comparisons between groups. The chi-square test was used for categorical data comparisons. Logistic regression models were used to correct for possible confounding variables, namely, prematurity, birth weight, and sedative therapy. Receiver operating characteristics (ROC) curve analysis was performed to assess the accuracy of bandwidth in predicting short-term neurological outcome.

## 3. Results

### 3.1. Description of the Sample

We considered 68 neonates admitted to our ICU between December 2023 and September 2024. From this sample, thirteen neonates were excluded: eight did not undergo surgery (two died preoperatively and six were deemed ineligible due to high surgical risk), one died within hours after surgery before monitoring could begin, one did not undergo monitoring due to a technical error, and three underwent monitoring of insufficient duration (<11 h). The final cohort included 55 neonates (31 males, 56.4%; 24 females, 43.6%) who underwent postoperative aEEG/cEEG monitoring for at least 11 h. Demographic and clinical data are summarized in Table 1.

Seizures were observed in 5/55 (9%) patients. In 1/55 seizures occurred at T0, while in the remaining four, seizures were recorded during extra protocol recording. Three neonates progressed from recurrent seizures to status epilepticus, one exhibited an isolated seizure that progressed to recurrent seizures, and one had recurrent seizures only. In the three neonates with status epilepticus, seizures occurred during additional monitoring following ECMO placement in the days after cardiac surgery. The neonate with recurrent seizures was diagnosed upon arrival in the ICU during the preoperative period, after abnormal clonic movements were observed in the left upper limb. This patient was the only one to also show clinical phenomenology associated with the detected electrical seizures. Finally, in the child who presented isolated seizures tending to evolve into recurrent ones, only electrical seizures were identified in an extra monitoring performed due to the overall critical clinical status. All five patients underwent neuroimaging (brain magnetic resonance imaging, MRI, and computerized tomography, CT), which revealed ischemic lesions in each case. Among the 55 included patients, 5 had genetically confirmed syndromes (9%), namely two DiGeorge syndrome (22q11.2 deletion), two trisomy 21, and one Shone’s syndrome. All patients underwent the post-surgical therapeutic regime routinely used in our center, including combinations of fentanyl (2 mcg/kg/h), midazolam (0.2 mg/kg/h), and rocuronium (0.01 mg/kg/h) if the sternum was maintained open after surgery or morphine (10 mcg/kg/h) and midazolam (0.2 mg/kg/h) if the sternum was closed right after surgical correction. In these patients, midazolam administration may have masked the potential onset of seizures. At discharge, neurological outcomes were normal in 37 patients (67.30%) and abnormal in 18 patients (32.72%), including 12 (21.81%) with moderately altered and six (10.9%) with severely altered neurological states (including two severe outcomes and four deaths).

### 3.2. aEEG Patterns and Trends

The classifications of aEEG recordings and their relative frequencies are presented in Table 2. Forty-four T0 recordings were available. At baseline, 31 neonates (56.36%) had normal aEEG, and 13 had moderately abnormal tracings. There were no severely abnormal tracings at baseline. For all patients, T1 and T2 recordings were available. After surgery, during monitoring, only two neonates showed normal aEEG, while the majority of them ranged from moderately abnormal plus to moderately abnormal minus. At T1, 44 patients (80%) remained stable (not improved aEEG), while 11 (20%) showed improvement in the EEG pattern (improved aEEG).

At T2, 40 (72.72%) patients had normal aEEG, 13 (23.63%) had moderately abnormal plus, and two (3.63%) had moderately abnormal minus. Across all the time points, none of the patients had severely abnormal aEEG. None of the patients with abnormal aEEG at T0 showed improvement in the trend during monitoring (T1), whereas 8 of 31 patients (25.8%) with normal aEEG at T0 exhibited improvement across all three aEEG monitoring segments performed at T1 after surgery. Chi-squared tests did not find significant associations between neurological outcome at ICU discharge and aEEG classification at T0 (chi-square = 4.1, *p* = 0.082) or T1 (segment A: chi-square = 4.45, *p* = 0.108; segment B: chi-square = 2.55, *p* = 0.280; segment C: Pearson chi-square = 2.58, *p* = 0.274). Conversely, classification at T2 was associated with short-term neurological outcome (chi-square = 17.69, *p* < 0.001), with 30 (90.90%) of the patients with a normal neurological outcome having normal aEEG at T2, while 11 (64.70%) of the patients with an abnormal neurological outcome presented a moderately abnormal aEEG. These associations still remained significant after adjusting for gestational age and ongoing medications (*p* = 0.025).

### 3.3. Association Between aEEG Parameters and Clinical Variables

**Figure 2:**
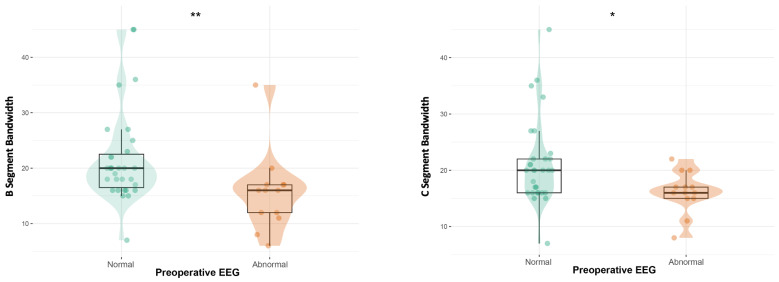
B-segment aEEG bandwidth according to preoperative EEG status (T0); C-segment aEEG bandwidth according to preoperative EEG status (T0). * = *p* < 0.05; ** = *p* < 0.01.

Patients with normal preoperative (T0) aEEG displayed a wider bandwidth in segments B (*p* = 0.004) and C (*p* = 0.018) than patients with an abnormal pre-surgery EEG. After adjusting for ongoing medication, the difference in bandwidth is confirmed only in C (*p* = 0.049) (Figure 2).

**Figure 3:**
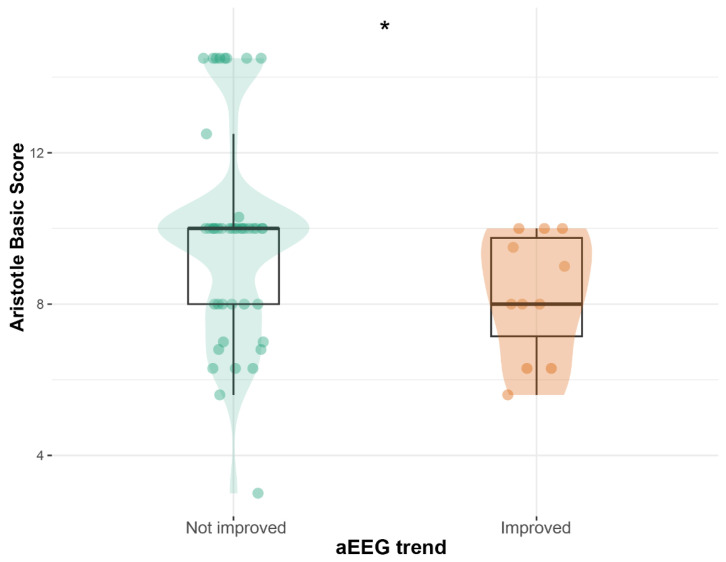
Aristotle basic score according to aEEG monitoring trend (T1). * = *p* < 0.05.

We compared patients who had improved aEEG (11/55) and those who had not improved aEEG (44/55) during T1. There were no differences between groups regarding demographic variables. Patients with improved aEEG trends had lower Aristotle basic scores compared with those who did not improve (8 [IQR = 1] vs. 10 [IQR = 2]; *p* = 0.040; Figure 3).

### 3.4. Association Between aEEG Parameters and Neurological Outcome

**Figure 4:**
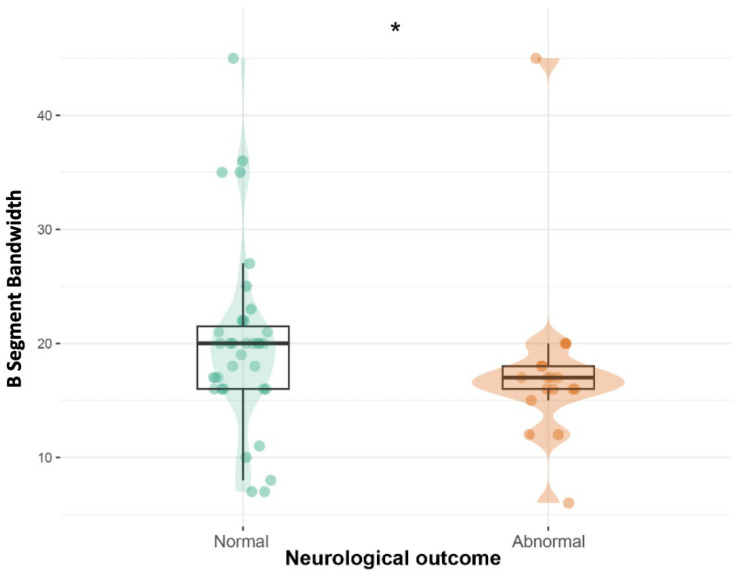
B-segment aEEG bandwidth according to neurological outcome. * = *p* < 0.05.

We compared patients who had normal (37/55) and abnormal (18/55) neurological outcomes at discharge. There were no differences between groups regarding demographic and clinical variables. We found that patients with abnormal neurological worsening had narrower B bandwidth (*p* = 0.041; Figure 4).

**Figure 5:**
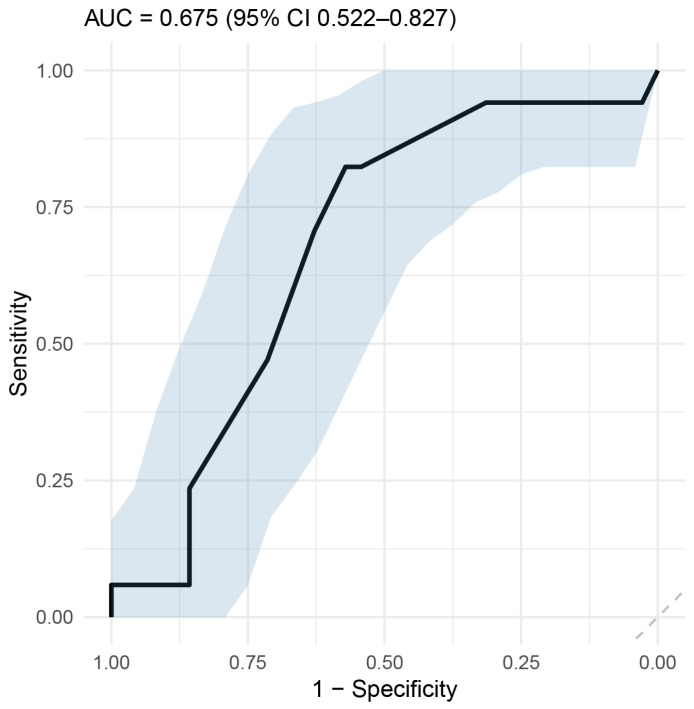
ROC curve for B-segment aEEG amplitude predicting neurological outcome. Light-blue shaded areas represent the confidence interval.

Considering this difference, we use an ROC curve analysis to assess the accuracy of the B segment amplitude in predicting neurological outcome. We found that a threshold of 17.5 µV predicted the neurological outcome with 70.6% sensitivity and 62.9% specificity, corresponding to a negative predictive value of 81.48% for adverse neurological outcomes (Figure 5).

## 4. Discussion

In this study, we investigated a relatively large monocentric cohort of neonates with CHD undergoing combined cEEG/aEEG monitoring, with the primary objective of deriving clinically meaningful prognostic indices from aEEG background activity. Although aEEG classification was associated with short-term neurological outcome only at T2, our segmented analysis of early postoperative aEEG revealed consistent and clinically relevant patterns. Neonates showing progressive improvement in aEEG during T1 had lower Aristotle scores, suggesting that reduced surgical and disease complexity is associated with more favorable neurophysiological trajectories. This improvement was also strongly linked to baseline (T0) aEEG features: infants with postoperative improvement consistently presented normal preoperative aEEG and wider bandwidths during T1, particularly in later segments. After adjustment for sedative therapy, these differences remained significant, supporting a physiological rather than purely pharmacological explanation. Conversely, neonates with abnormal preoperative aEEG did not exhibit postoperative improvement. Moreover, reduced amplitude margins and narrower bandwidth during intermediate monitoring (segment B) were associated with worse neurological outcomes at ICU discharge. From a pathophysiological perspective, these features likely reflect impaired organization of cerebral activity, reduced variability, and absent cyclicity, all indicative of disrupted neural network function.

These findings, albeit preliminary, suggest that early postoperative aEEG background assessment may represent a valuable adjunct for preoperative and early postoperative counseling with parents. We focused on prolonged aEEG monitoring performed within the first 24–48 h after cardiac surgery, a critical time window during which sedative therapy was still ongoing. Our results demonstrated that early post-surgical aEEG background is typically characterized by reduced amplitude and loss of normal physiological cyclicity, findings that likely reflect the combined effects of sedative medications and early postoperative cerebral vulnerability. Importantly, however, these alterations may also represent early markers of cerebral distress during the immediate postoperative period [23].

The ability to identify neonates at increased risk of adverse neurological outcomes based on early neurophysiological markers could support more informed prognostic discussions and individualized clinical decision-making.

The complexity of the underlying cardiac condition, as quantified by the Aristotle basic score, emerged as a key determinant of both surgical burden and neurophysiological evolution. Higher Aristotle scores reflect not only greater procedural complexity but also the intrinsic severity of the underlying cardiac malformation. In clinical practice, more complex CHD and surgical procedures are often associated with longer cardiopulmonary bypass times, aortic cross-clamping, and, in selected cases, circulatory arrest, all of which may increase the risk of perioperative cerebral hypoperfusion and neurological vulnerability [24]. In this light, the association between lower Aristotle scores and more favorable aEEG trajectories may support the hypothesis that reduced surgical and anatomical complexity translates into lower perioperative cerebral stress. Nevertheless, further studies are needed to clarify the independent contribution of specific CHD subtypes (for example, cyanotic versus acyanotic lesions) to postoperative aEEG patterns and neurological outcomes.

These factors are well-recognized contributors to perioperative brain injury [2], underscoring the clinical relevance of systematic neurophysiological monitoring in this particularly vulnerable population [1].

To date, seizures have been regarded as the principal indication for prolonged EEG monitoring in neonates with CHD [1,2,25,26,27], with cEEG considered the gold standard. While aEEG has well-recognized limitations in seizure detection [28], parameters beyond ictal activity—particularly aEEG systematic background assessment—may provide additional and clinically relevant prognostic information regarding neurological status and recovery, in particular for populations such as hypoxic–ischemic encephalopathy patients [29]. Seizures were observed in five neonates (9%), in line with prevalence rates reported in the literature [2]. Notably, no seizures occurred during the standard immediate postoperative monitoring window. Instead, seizures were detected either preoperatively in a clinically unstable neonate (one case) or later in the post-surgical phase (four cases), all in the context of critical illness, including two cases during extracorporeal membrane oxygenation (ECMO). Only one neonate exhibited electroclinical seizures, while the remaining cases involved exclusively electrographic events. Seizure occurrence was associated with poor short-term neurological outcomes: three infants developed major impairments in alertness, tone, and motor function (two severe and one moderate), and two infants died within weeks of seizure onset. Although statistical analysis was not feasible due to the limited sample size, these observations further emphasize the clinical value of neurophysiological monitoring for seizure detection and management in critically ill neonates, as previously highlighted [25,26].

Overall, beyond its role in seizure and status epilepticus detection, aEEG—through systematic background analysis—may provide valuable real-time insights into cerebral functional status in neonates. Nevertheless, aEEG presents important limitations [28]. Compared with cEEG, it does not offer detailed spectral information, fails to capture neonatal physiological graphoelements (such as anterior sharp transients, anterior slow dysrhythmia, or positive central/temporal/rolandic spikes), and provides limited spatial resolution of epileptiform activity. For these reasons, aEEG should be regarded as a complementary modality rather than a substitute for conventional EEG [26,27]. A combined approach integrating both aEEG and cEEG monitoring may therefore represent the optimal standard of care for neurophysiological assessment in critically ill neonates [15,28,30].

Our findings align with and further extend the existing body of literature supporting the role of aEEG as a reliable bedside tool for neuromonitoring and early prognostic stratification in neonates with CHD. Previous studies [31,32,33] have consistently demonstrated that abnormalities in aEEG background activity, particularly discontinuity, burst suppression, and absence or delayed recovery of sleep–wake cycling, are associated with both structural brain injury and adverse neurodevelopmental outcomes. In a prospective cohort, Latal et al. [31] showed that abnormal postoperative aEEG background patterns and delayed reappearance of sleep–wake cycles independently predicted lower cognitive performance at 4 years, underscoring the long-term prognostic relevance of early electrophysiological alterations. Similarly, Mulkey et al. [32] reported a strong association between early abnormal aEEG patterns and preoperative brain injury on MRI, suggesting that aEEG may serve as a surrogate marker of underlying structural vulnerability already present in the immediate neonatal period. Consistent with these findings, early postnatal studies have shown that abnormal aEEG background activity is frequent in CHD neonates and may be influenced by both prenatal factors and perioperative exposures, including sedation and hemodynamic instability [33]. Within this framework, our results reinforce the concept that aEEG-derived measures of background activity are not merely descriptive but carry clinically meaningful information regarding brain function and vulnerability. Importantly, by focusing on dynamic changes in aEEG bandwidth and trend evolution in the early postoperative phase, our study adds a temporal dimension to previous observations, suggesting that not only static patterns but also their trajectory over time may provide incremental prognostic value.

This study has several limitations. Its real-world design inherently constrained sample size and data completeness. Of the 68 eligible neonates, only 55 underwent aEEG monitoring due to urgent surgical interventions, limited availability of neurophysiology personnel, or exclusion for severe cardiorespiratory instability. Complete longitudinal data across all time points (T0, T1, and T2) were available for only 44 neonates, as early surgical scheduling precluded baseline recordings in some cases. Neuroimaging (MRI or CT) was obtained in only a subset of patients, primarily those with significant clinical concerns. Furthermore, follow-up was restricted to the end of the neonatal period or ICU discharge, as many patients were transferred back to referring centers shortly after stabilization, limiting assessment of long-term neurological outcomes.

Although seizures were not the primary focus of the present study and the prevalence observed in our cohort is consistent with the previous literature [1,2,6,7,8], it should be acknowledged that most seizures were identified during extra-protocol recordings. Therefore, the limited duration of protocolized monitoring may have led to an underestimation of seizure occurrence in the early postoperative phase.

A further limitation, inherent to the real-world design of the study, relates to the comparability of T1 recordings. Although all recordings were performed within a defined early postoperative window (24–48 h), variability in recording onset and duration may have introduced heterogeneity across patients, potentially limiting strict inter-subject comparability.

Moreover, medications’ effects on the EEG tracing, despite a certain uniformity in the treatment within our cohort, may differentially affect each subject and confound the outcomes. Finally, to classify aEEG, we used an in-house-designed system based on the previous literature. While this limitation may affect the generalizability and comparability of the study, it allowed us to capture subtle background trends, including bandwidth variations, along the recording, especially in prolonged monitoring sessions. With our classification system, we tried to apply a more nuanced description of the monitoring trends in the postoperative periods, reintroducing the concept of bandwidth previously introduced in the Burdjalov classification [16]

Despite these limitations, the real-world nature of the study accurately reflects routine clinical practice in neonatal and pediatric intensive care units using aEEG, where resource and staffing constraints may hinder strict adherence to standardized monitoring protocols recommended by international guidelines [14,34], which often advocate prolonged recordings of up to 24 h with cEEG. Importantly, all data were retrospectively collected from neonates receiving standard clinical care rather than from an interventional research protocol, enhancing the external validity and generalizability of our findings. In this context, real-world constraints underscore the need for rationalization of neurophysiological resources and support the clinical utility of targeted, prognostically informative aEEG monitoring strategies.

## 5. Conclusions

Neonates undergoing surgical intervention for CHD are highly vulnerable to both perioperative and postoperative neurological complications. Early and continuous assessment of brain function is essential to identify subclinical injury, guide clinical management, and inform prognostic trajectories. Our study highlights the value of multimodal neuromonitoring in this vulnerable population. By integrating cEEG with aEEG, this approach captures complementary aspects of cerebral activity, including both global brain function and transient pathological events, such as seizures. aEEG signal parameters provide crucial insights into cerebral health and may serve as early indicators of neurological outcomes. aEEG remains a useful tool for monitoring critically ill neonates, especially when utilized alongside traditional EEG. While no single modality can fully predict long-term neurodevelopmental outcomes, the use of structured, combined monitoring significantly enhances the detection of functional brain alterations and enables timely, targeted intervention. Overall, this approach represents a critical component of neurocritical care for neonates with CHD, improving early, individualized management and supporting the optimization of long-term neurological health. Further studies with larger cohorts and longer follow-up are necessary to fully elucidate the role of aEEG trends in predicting long-term neurodevelopmental outcomes in neonates with surgically treated CHD.

## Figures and Tables

**Table 1:** Demographic and clinical data.

Variables	Frequencies (%)
**Sex**	
Male	31 (56.40)
Female	24 (43.60)
**Gestational Age (ranges)**	
28–31	1 (1.80)
32–36	6 (10.90)
>/=37	48 (87.27)
**Delivery**	
Spontaneous	23 (41.80)
Cesarean	29 (52.70)
ND	3 (5.45)
**Neonatal weight (NW)**	
>2500 g	39(70.90)
1500–2499 g	12 (21.81)
1000–1499 g	1 (1.80)
<1000 g	1 (1.80)
ND	2 (3.60)
**APGAR (1st min)**	
8–10	35 (63.60)
4–7	9 (16.40)
0–3	4 (7.30)
ND	7 (12.72)
**APGAR (5th min)**	
8–10	36 (65.45)
4–7	6 (10.90)
0–3	1 (1.80)
ND	12 (21.80)
**APGAR (10th min)**	
8–10	3 (5.45)
4–7	3 (5.45)
0–3	0
ND	49 (89.10)
**AGA/SGA**	
AGA	45 (81.81)
SGA	8 (14.54)
ND	2 (3.60)
**Neurological outcome**	
Normal	37 (67.30)
Abnormal	18 (32.72)
Moderately altered	12 (21.81)
Severely altered	6 (10.90)
Deceased	4 (7.30)

Abbreviations: ND, not determinable; AGA, appropriate for gestational age; SGA, small for gestational age.

**Table 2:** aEEG recording classifications.

aEEG Recordings	Frequencies (%)
**T0**	
Normal	31 (56.36)
Moderately Abnormal Plus	9 (16.36)
Moderately Abnormal Minus	4 (7.27)
Severely Abnormal	0
Not Performed	11 (20)
**T1—Segment A**	
Normal	2 (3.64)
Moderately Abnormal Plus	16 (29.09)
Moderately Abnormal Minus	37 (67.27)
Severely Abnormal	0
Not Performed	0
**T1—Segment B**	
Normal	3 (5.45)
Moderately Abnormal Plus	23 (41.82)
Moderately Abnormal Minus	29 (52.73)
Severely Abnormal	0
Not Performed	0
**T1—Segment C**	
Normal	3 (5.45)
Moderately Abnormal Plus	25 (45.45)
Moderately Abnormal Minus	27 (49.09)
Severely Abnormal	0
Not Performed	0
**T2**	
Normal	40 (72.72)
Moderately Abnormal Plus	13 (23.63)
Moderately Abnormal Minus	2 (3.63)
Severely Abnormal	0
Not Performed	0

## Data Availability

All data necessary to evaluate the current work are available within the manuscript and Supplementary Material. Further data might be requested from the corresponding author by qualified researchers and/or institutions.

## Supplementary Materials

The following supporting information can be downloaded at https://www.mdpi.com/article/10.3390/neurosci7020048/s1. Table S1: A brief summary of the classification used in the article.

## Abbreviations

The following abbreviations are used in this manuscript:

CHD	congenital heart disease
EEG	electroencephalography
cEEG	conventional electroencephalography
aEEG	amplitude-integrated electroencephalography
ICU	intensive care unit
GA	gestational age
AGA	appropriate for gestational age
SGA	small for gestational age
ECMO	extracorporeal membrane oxygenation
PNG	pneumogram
EOG	electro-oculogram
EMG	electromyogram
IQR	interquartile ranges
MRI	magnetic resonance imaging
CT	computerized tomography
ND	not determinable
ECG	electrocardiogram
