# Supplementary material for: The Role of Background Activity Monitoring by Amplitude-Integrated EEG to Predict Short-Term Neurological Outcome in Neonates with Congenital Heart Disease: Insights from a Real-Life Retrospective Cohort
Source: NeuroSci. 2026 Apr 20;7(2):48. doi: 10.3390/neurosci7020048 (PMC13118592; doi:10.3390/neurosci7020048)
Supplement: Supplementary file 1 [file neurosci-07-00048-s001.zip › neurosci-4138277-supplementary.pdf]

Supplementary

Massimo Mastrangelo et al. **The Role of Background Activity Monitoring by Amplitude-Integrated EEG to Predict Short-Term Neurological Outcome in Neonates with Congenital Heart Disease: Insights from a Real-Life Retrospective Cohort**

Supplementary Materials

Materials used included: "Natus Brain Quick-Micromed" headbox with 34 recording channels; "SPES medica" silver/silver chloride (AgAgCl) cup electrodes for EEG and single-use surface electrodes for polygraphic recording; "Nuprep" abrasive skin-prep gel; "TEN20" conductive-adhesive paste; "Bendelast" elastic tubular net (sizes 5–6, depending on the patient's head circumference); Pneumogram (PNG) belt; "Natus Brain Quick-Micromed" video camera. Polygraphic channels were included to improve monitoring of sleep-wake cycles and potential epileptic seizures.

Supplementary Table S1 A brief summary of the classification used in the article

aEEG	Upper margin	Lower margin	Band	Ciclicity	Lower margin variability	Asimmetry > 50%	Seizure
Normal	> 10 µV	> 5 µV	< 15 µV, in active wake–sleep (with lower margin > a 5 µV) ≤ 50 µV, in quiet sleep (with lower margin > 5 µV)	Constantly present	yes	no	no
Moderately Abnormal Plus	≥ 10 µV	≤ to 5 µV	Not definable based on the literature	Initial / non-constant	Reduced, ≤5 µV	yes/no	yes/no
Moderately Abnormal Minus	> 10 µV	< 5 µV	Not definable based on the literature	Initial / non-constant	Absent or reduced, does not exceed 5µV	yes/no	yes/no
Severely Abnormal	< 10 µV	< 5 µV	< 15 µV, with lower margin < 5 µV; > 20 µV, with lower margin < 5 µV	no	no	yes/no	yes/no
